# *Beta protein 1* homeoprotein induces cell growth and estrogen-independent tumorigenesis by binding to the estrogen receptor in breast cancer

**DOI:** 10.18632/oncotarget.10633

**Published:** 2016-07-16

**Authors:** Sidney W. Fu, Saurabh P. Kirolikar, Erika Ginsburg, Xiaohui Tan, Arnold Schwartz, Samuel J. Simmens, Yan-Gao Man, Joseph J. Pinzone, Christine Teal, Sanket Awate, Barbara K. Vonderhaar, Patricia E. Berg

**Affiliations:** ^1^ Department of Medicine, Division of Genomic Medicine, George Washington University, Washington, DC 20037, USA; ^2^ Department of Biochemistry and Molecular Medicine, George Washington University, Washington, DC 20037, USA; ^3^ Mammary Biology and Tumorigenesis Laboratory, National Cancer Institute, National Institutes of Health, Bethesda, MD 20892, USA; ^4^ Department of Pathology, George Washington University Medical Center, Washington, DC 20037, USA; ^5^ Department of Epidemiology and Biostatistics, School of Public Health and Health Services, George Washington University, Washington, DC 20037, USA; ^6^ Department of Gynecologic and Breast Pathology, Armed Forces Institute of Pathology, Washington, DC 20306, USA; ^7^ David Geffen School of Medicine, UCLA, Los Angeles, CA 90095, USA; ^8^ Department of Surgery, George Washington University, Washington, DC 20037, USA

**Keywords:** homeobox gene, BP1, estrogen receptor, tamoxifen resistance, tumorigenesis

## Abstract

Expression of *Beta Protein 1 (BP1)*, a homeotic transcription factor, increases during breast cancer progression and may be associated with tumor aggressiveness. In our present work, we investigate the influence of *BP1* on breast tumor formation and size *in vitro* and *in vivo*. Cells overexpressing BP1 showed higher viability when grown in the absence of serum (*p* < 0.05), greater invasive potential (*p* < 0.05) and formed larger colonies (*p* < 0.004) compared with the controls. To determine the influence of BP1 overexpression on tumor characteristics, MCF-7 cells transfected with either empty vector (V1) or overexpressor plasmids (O2 and O4) were injected into the fat pads of athymic nude mice. Tumors grew larger in mice receiving O2 or O4 cells than in mice receiving V1 cells. Moreover, *BP1* mRNA expression levels were positively correlated with tumor size in patients (*p* = 0.01). Interestingly, 20% of mice injected with O2 or O4 cells developed tumors in the absence of estrogen, while no mice receiving V1 cells developed tumors. Several mechanisms of estrogen independent tumor formation related to BP1 were established. These data are consistent with the fact that expression of breast cancer anti-estrogen resistance 1 (*BCAR1*) was increased in O2 compared to V1 cells (*p* < 0.01). Importantly, O2 cells exhibited increased proliferation when treated with tamoxifen, while V1 cells showed growth inhibition. Overall, *BP1* overexpresssion in MCF-7 breast cancer cells leads to increased cell growth, estrogen-independent tumor formation, and increased proliferation. These findings suggest that BP1 may be an important biomarker and therapeutic target in *ER* positive breast cancer.

## INTRODUCTION

Beta Protein 1(*BP1*), an isoform of *DLX4*, belongs to the homeobox family of genes, master regulatory genes implicated in early development and cell differentiation that are frequently deregulated in cancer [1, 2]. Aberrant expression of *BP1* has been shown in women with breast cancer. *HOXB7*-transduced SkBr3 cell lines developed tumors in nude mice in the absence of irradiation, while control mice injected with SkBr3 cells did not form tumors under those conditions [3]. Importantly, repression of *HOXA5* in breast cancer resulted in the loss of expression of the tumor suppressor p53 [4]. Moreover, constitutive expression of *HOXA1* in MCF7 cells led to increased anchorage-independent growth and tumor formation in mice [5]. BP1 directly activates the anti-apoptotic gene *BCL-2* and results in resistance to *TNF-α*. In sporadic breast cancer, *BP1* negatively regulates the expression of breast cancer anti-estrogen resistance 1 (*BRCA1)* through binding to its intron, suggesting that overexpression of *BP1* might be a potential inhibitor of *BRCA1*. Therefore, targeting *BP1* may provide a new avenue for breast cancer management [6].

*BP1* belongs to the Distal-less subfamily of the homeobox gene family [7]. In our earlier study, *BP1* expression was examined in untreated invasive ductal breast carcinoma (IDC) using semi-quantitative RT-PCR [8]. Overall, *BP1* mRNA expression was found in 80% of breast tumors, with an 11% rate of low *BP1* mRNA in normal tissues, while 100% of ER-negative tumors expressed *BP1*. These data suggest *BP1* might be a useful target for therapy in patients with ER-negative tumors. In a follow-up study, we examined IDC cases from the Armed Forces Institute of Pathology which included women from around the world [9]. Eighty one percent of invasive ductal carcinomas were *BP1* positive by immunostaining, indicating excellent agreement between BP1 RNA expression (80%) and protein expression (81%).

Estrogens are crucial hormones involved not only in normal breast development but also in carcinogenesis of breast epithelium and progression of breast cancer [10]. Estrogens act through a specific receptor, the estrogen receptor (ER). Once activated by estrogen, ER forms a transcriptional complex with various co-activators and co-repressors on target gene promoters to regulate their expression [11] ER-negative breast cancers are unresponsive to anti-estrogen therapy. In general, these tumors have a higher histologic grade and a higher proliferative rate and are associated with poorer prognosis. In this paper we found estrogen independence associated with high BP1 expression in ER-positive tumors in cell lines and in mice. To determine the molecular mechanism that contributes in part or in whole to *BP1*-related breast cancer aggressiveness in ER+ tumors, *in vitro* experiments were carried out. Here we present a novel model of *ER* regulation and estrogen independence by *BP1*.

## RESULTS

### Increased levels of *BP1* are associated with a more aggressive phenotype in MCF-7 cells *in vitro*

**Figure 1:**
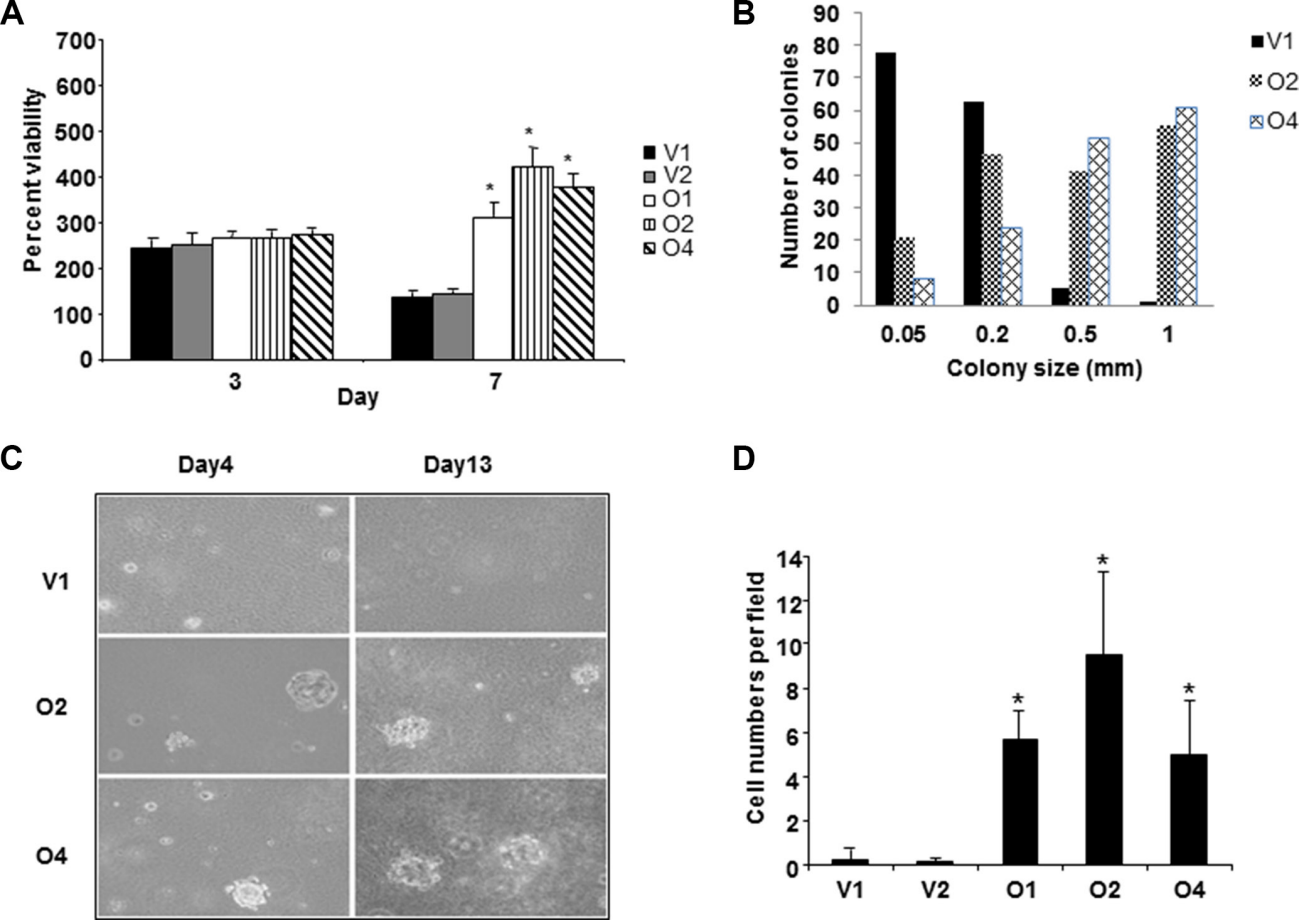
Overexpression of *BP1* in MCF-7 cells is associated with aggressiveness (**A**) Growth in the absence of serum. V1, O2 and O4 cell lines were grown without serum for seven days and cell number was measured using MTT assays. Data was normalized against day 0, which was set as 100%. Asterisks denote a statistical significance of *p* < 0.05 as compared to V1. (**B**) Colony formation in soft agar. V1, O2 and O4 cell lines were grown in soft agar as described in Materials and Methods. (**C**) Photomicrographs of cells grown in soft agar for four or 13 days. (**D**) Invasion of cells through Matrigel. *BP1* overexpressing cell lines (O2 and O4) exhibited higher invasiveness compared to the empty vector control cell lines (V1) (*p* < 0.05). Three fields of unit area on each membrane were counted for cell numbers and the experiments were repeated twice.

MCF-7 cells containing an empty vector (V1) or a plasmid stably overexpressing *BP1* cDNA under control of the CMV promoter (O2 and O4) were tested using classical assays which assess the oncogenic characteristics of cells. These cell lines were previously shown to express increased levels of *BP1* mRNA and protein [12]. In the first assay, cells were grown in the absence of serum to test growth factor independence. By day seven in the absence of serum (Figure 1A), cell lines overexpressing *BP1* (O2 and O4) showed approximately two to three-fold higher viability compared with V1, a statistically significant difference (*p* < 0.05). These data suggest that high *BP1* levels may protect against cell death in the absence of serum, consistent with the increased *BCL-2* expression in *BP1*-overexpressing MCF-cells, as reported [12].

Growth in soft agar was utilized to determine the anchorage independent growth of *BP1*- transfected MCF- 7 cells. This assay is considered a reliable method for detecting the malignant potential of cells [13]. While the total number of colonies was similar for the cell lines (V1 = 150, O2 = 161, O4 = 152), the cells overexpressing *BP1* produced larger and more rapidly growing colonies (Figure 1B and 1C). Ninety-five percent of the colonies formed by V1 cells were equal to or less than 0.2 mm, while 88–93% of O2 and O4 colonies were greater than 0.2 mm. Both the O2 and the O4 cell lines had a significantly higher distribution of colony sizes compared to the V1 cell lines (*p* < 0.0001, Wilcoxon Rank Sum test). Even by day 4, the colonies derived from O2 and O4 were noticeably larger than those from V1 (Figure 1C).

Whereas MCF-7 cells are poorly invasive through Matrigel [14], we wished to determine whether *BP1* could modulate their invasiveness. As shown in Figure 1D, O2 and O4 cells overexpressing *BP1* increased invasion of MCF-7 cells by approximately 16-fold compared with control V1 cells, which was statistically significant (*p* < 0.05). These data suggest that *BP1* levels may affect the metastatic potential of breast cancer, as previously demonstrated in ER-negative Hs578T breast cancer cells [15].

### Characteristics of tumor growth in mice

**Figure 2:**
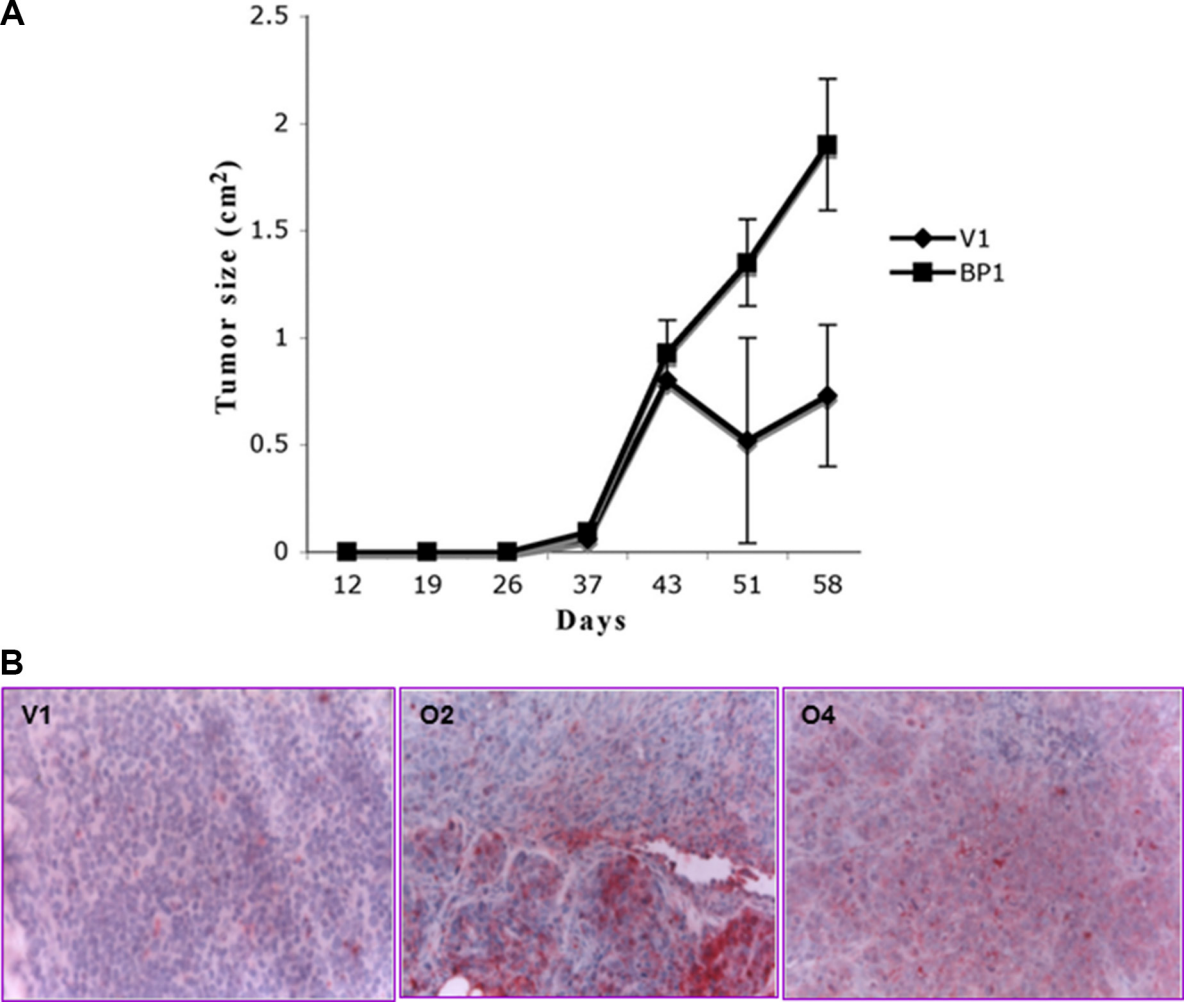
Tumor growth in mice is enhanced by increased BP1 expression (**A**) Tumor size. Nude mice supplemented with a cholesterol-based estrogen pellet were injected in the cleared mammary fat pad with V1, O2 or O4 cell lines. Tumors were measured twice weekly and allowed to grow up to 58 days. (**B**) Immunostaining of tumors with anti-*BP1* antibody. Anti-*BP1* antibody was used to immunostain tumor sections. Since no tumors derived from V1 cells formed in the absence of an estrogen supplement, all sections are from estrogen supplemented mice. Figures are shown at 300X magnification.

It has previously been established that MCF-7 cells grow as a solid tumor when placed in the mammary glands of mice supplemented with estrogen [16]. To determine whether high *BP1* levels would influence tumor characteristics, we injected V1, O2 or O4 cells into cleared fat pads of athymic nude mice, with 10 mice per group. Palpable tumors were present at 45 days post-surgery in the mammary glands of mice supplemented with an estrogen pellet (Figure 2A). Due to the relatively small number of mice, data from mice injected with O2 and O4 cells were combined. Tumors derived from O2 and O4 cells, labeled *BP1* (Figure 2A), appeared to grow at a faster rate and were larger by day 58 than tumors from control V1 cells, but the small number of tumors precluded significance testing. No distant metastases were observed for any mice at the time of sacrifice. *BP1* expression was retained in the tumors, shown by protein expression analysis. In mice supplemented with estrogen, *BP1* expression was higher in tumors derived from O2 and O4 cells than in tumors derived from V1 cells (Figure 2B, red staining cells).

### Human breast tumor size correlates with *BP1* expression

**Figure 3:**
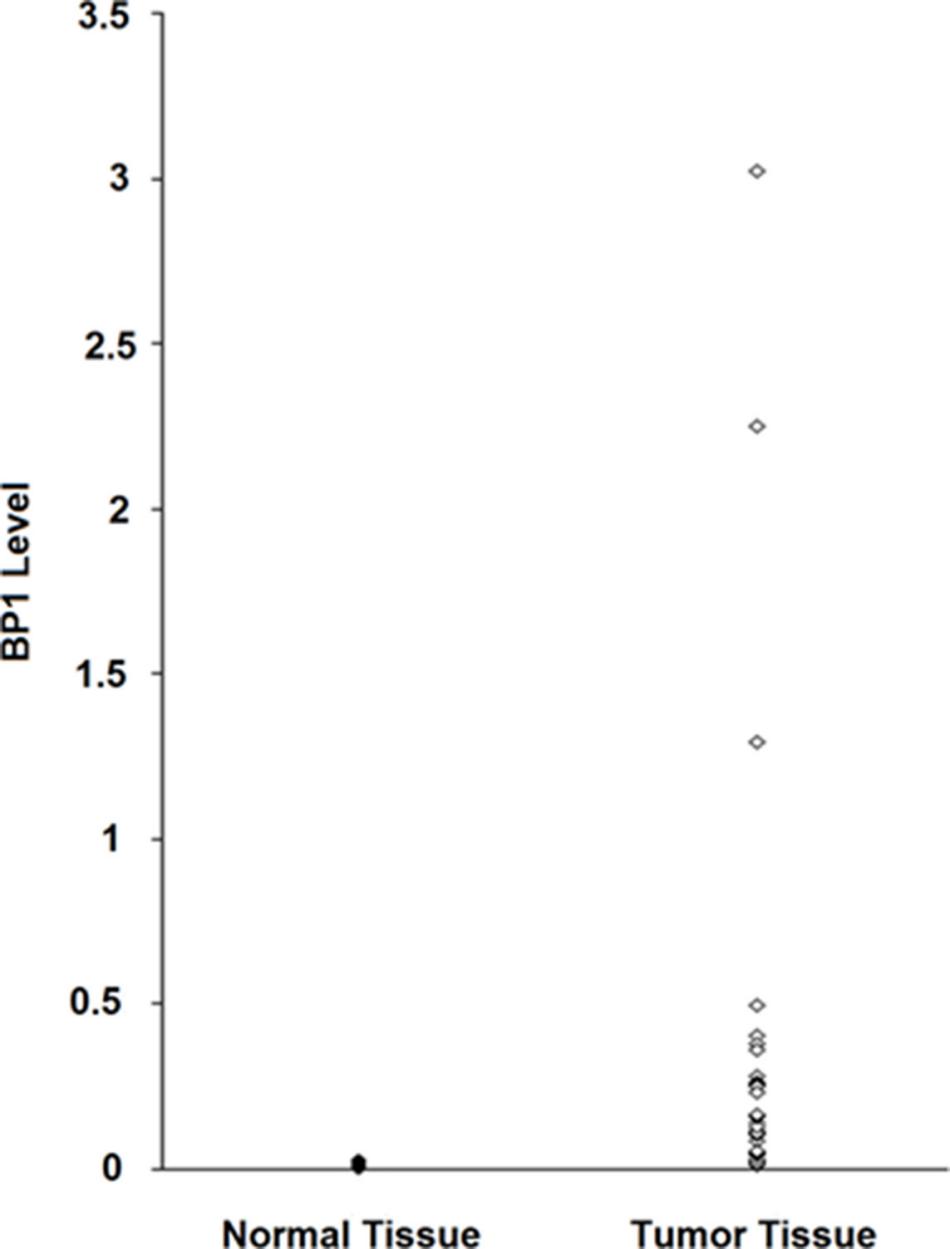
*BP1* expression levels in breast tumors RNA was extracted from 31 frozen tumors, followed by real-time PCR analysis. *BP1* levels were normalized to *18S* RNA. For comparison, RNA was extracted from five normal frozen breast tissues.

The possible clinical relevance of *BP1* expression level with respect to tumor size in breast cancer patients was determined. Real-time PCR was used to measure the levels of expression of *BP1* mRNA from 31 tumor samples. The levels of *BP1* expression, relative to *18S* RNA, ranged over 300-fold (Figure 3). For comparison, RNA was extracted from five normal breast tissues; *BP1* levels ranged from 0.010 to 0.060 relative to *18S* RNA. We found a positive correlation (*r* = 0.40, *p* = 0.02) between [log] *BP1* expression and [log] tumor size. After statistically controlling for age and race, the partial correlation coefficient remained statistically significant (partial *r* = 0.49, *p* = 0.01). Thus, there is an association between higher *BP1* mRNA expression levels and tumor size in patients.

### *BP1* expression is associated with estrogen independence

In general, MCF-7 cells require exogenous estrogen to form tumors in nude mice [16]. However, approximately 20% of the mice implanted with cells stably transfected with *BP1* (O2 or O4) were able to form tumors in the absence of estrogen, in contrast to 0% of mice injected with V1 cells (Table 1), but this difference was not statistically significant, probably due small sample size. Thus, *BP1* overexpression in MCF-7 cells may induce estrogen-independent tumorigenesis in mice.

**Table 1:** Estrogen dependence of mammary tumor formation in mice correlates with BP1 expression

Cell Type Injected	With Estrogen	Without Estrogen
V1	4/10 (40%)	0/10 (0%)
O2	10/15 (67%)	2/10 (20%)
O4	7/11 (64%)	2/10 (20%)

MCF-7 cell lines O2 and O4, which overexpress *BP1*, were compared with V1, a cell line containing the empty vector. The percentage of mice in each category is shown in parentheses.

### *BP1* regulates *ER via* two mechanisms

### Direct regulation

**Figure 4:**
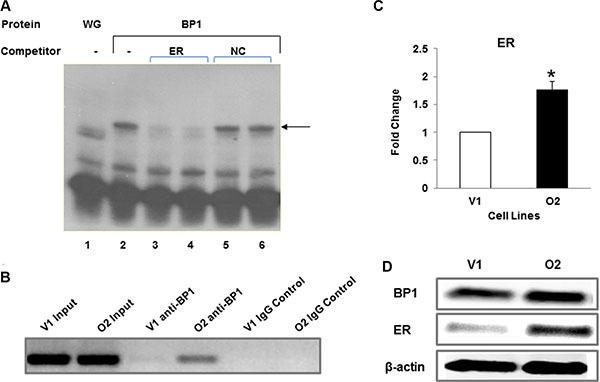
*BP1* binds to and regulates *ER* (**A**) Binding of BP1 to IVS1 of the estrogen receptor. Electrophoretic mobility shift assays were performed to detect potential binding of *in vitro* transcribed and translated *BP1* to a consensus binding sequence located in IVS1 of the estrogen receptor. Binding of *BP1* to a ^32^P end-labeled dsDNA probe containing the putative *BP1* binding site and surrounding sequence is observed as a shifted band (arrow). Lane 1, wheat germ extract (WG) alone incubated with the probe. Lane 2, *BP1* incubated with the probe. Lanes 3 and 4, unlabeled *ER* DNA (*ER*) was added to the incubation mixture containing *BP1* protein at 500 X or 1000X molar excess, respectively. Lanes 5 and 6, a nonspecific negative control (NC) DNA to which *BP1* protein does not bind was added at a 500 X or 1000X molar excess. (**B**) ChIP assay. DNA was precipitated using either the *BP1* antibody (anti-*BP1*) or IgG negative control (IgG control) and whole DNA as positive control (input). The quantity of IVS1 DNA precipitated by *BP1* antibody was compared in *BP1* overexpressing cells (O2) and the empty vector (V1). (**C**) qRT-PCR. mRNA level of *ER* was measured by qRT-PCR comparing empty vector (V1) and *BP1* overexpressor (O2) (*p < 0.01). Experiments were performed in triplicate. (**D**) Western blot analysis. Immunoblotting was performed with protein extracts from V1 and O2 cells as described in Materials and Methods.

A computer search revealed a consensus *BP1* binding site [7] located in the first intron (IVS1) of the *ER* gene. An electrophoretic mobility shift assay (EMSA) verified that *BP1* binds to this site *in vitro*. The consensus-binding site within a short stretch of IVS1 was used as a probe. As shown in Figure 4A, *BP1* binds to the probe (lane 2, arrow). The shifted band is specific, demonstrated by competition with the unlabeled probe DNA (lanes 3 and 4) but not with non-specific DNA (lanes 5 and 6). A ChIP assay was performed to validate the EMSA results. DNA was precipitated using the *BP1* antibody (anti-*BP1*) with O2 DNA or whole DNA (input) as positive controls, but not the IgG negative control (IgG control) (Figure 4B). The relative quantity of IVS1 precipitated by *BP1* antibody is much higher in O2 cells than in V1 cells. The qRT-PCR data (Figure 4C, *p < 0.01) and Western blot analysis (Figure 4D) suggest that binding of *BP1* transcriptionally upregulates ER.

### Indirect regulation

Previously, we showed that *BP1* negatively regulates *BRCA1* [6]. *P300* is a known histone acetyl transferase (HAT) protein which shares a dynamic relationship with *BRCA1* and *ER* [17, 18]. This led us to investigate the relationship between *BP1* and *p300*. We found a putative *BP1* binding region in the first intron of the *EP300* gene.

**Figure 5:**
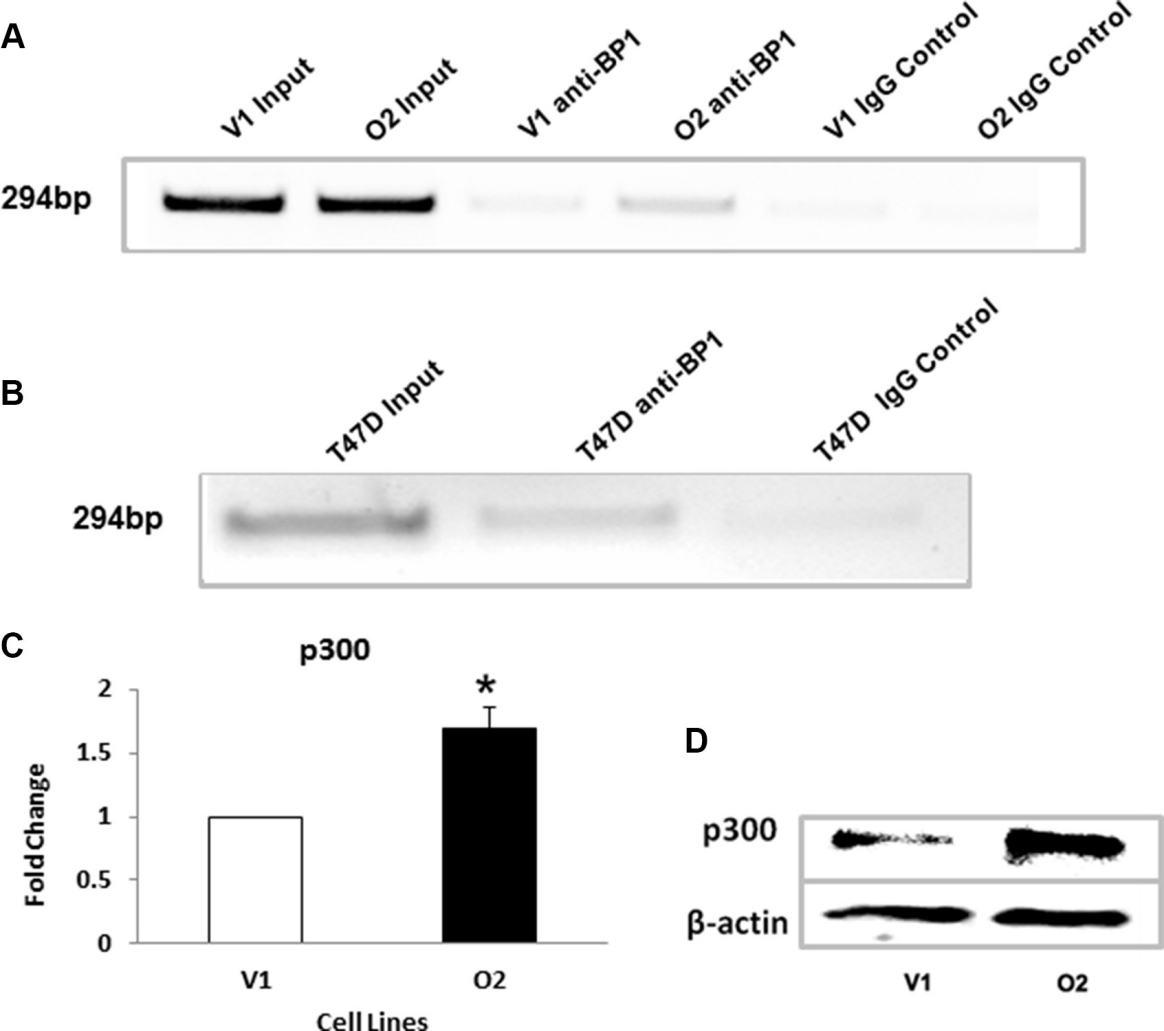
*BP1* binds to and upregulates *EP300* (**A, B**) ChIP assays. Chromatin immunoprecipitation was performed as described above to verify the binding of BP1 to the first intron of the *EP300* gene in MCF-7 cell derivatives and T47D cell lines, respectively. (**C**) qRT-PCR. mRNA levels of *p300* were measured by qRT-PCR comparing MCF-7 cells containing an empty vector (V1) and cells overexpressing *BP1* (O2) (*p < 0.05). Experiments were performed in triplicate. (**D**) Western blot analysis. Immunoblotting was performed to determine the levels of *p300* protein expression in V1 and O2 cells.

A ChIP assay was performed to verify the binding of *BP1* to the first intron of *P300*. Figure 5A and 5B show that BP1 binds to the *P300* gene in the first intronic region in MCF-7 cell derivatives and T47D cell lines, respectively. T47D cells are *ER*+ cell lines that have high endogenous levels of *BP1* proteins [8]. *BP1* binding is significantly higher in O2 cells than in V1 cells (Figure 5A). The qRT-PCR data (Figure 5C, *p < 0.05) and Western blot analysis (Figure 5D) show increased expression of *p300* in cells overexpressing *BP1* (O2) compared to V1 cells. Each experiment was repeated at least three times. Thus, *BP1* transcriptionally upregulates *P300*.

### Increased levels of *BP1* expression are associated with tamoxifen resistance

**Figure 6:**
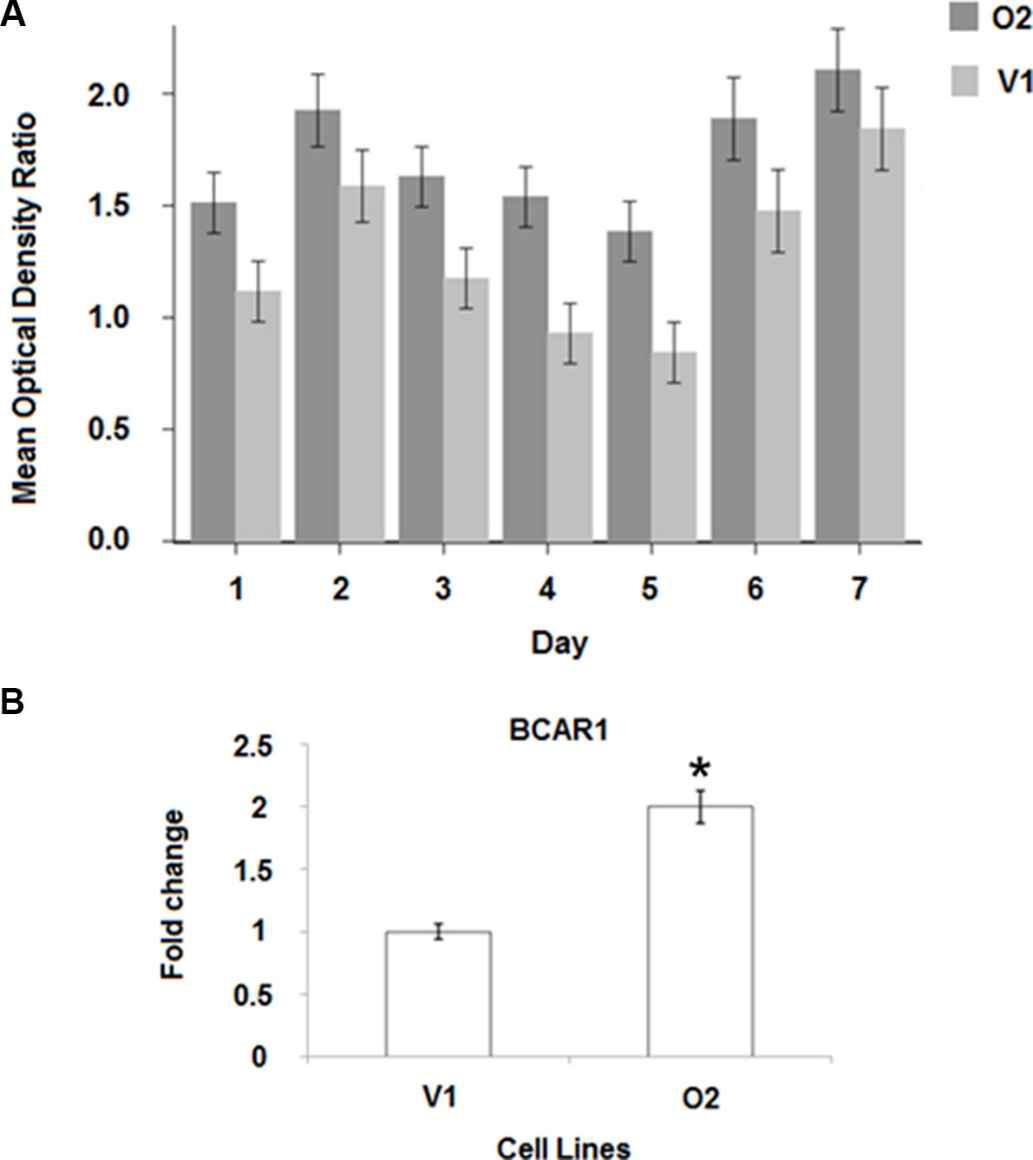
*BP1* increases tamoxifen resistance (**A**) MTT assay. V1 and O2 cells were challenged with 3 uM tamoxifen over seven days, as described in Materials and Methods. The 95% confidence limits are shown on the adjusted means. (**B**) mRNA levels of the tamoxifen resistance marker, *BCAR1* (breast cancer anti-resistance). *BP1* O2 cells have increased levels of *BCAR1* mRNA compared with V1 cells (*p < 0.01).

Our *in vivo* experiments indicate that *BP1* may be a contributing factor to estrogen independence. Consistent with this, we observed elevated levels of *ER* in O2 cells. To determine if increased *BP1* expression imparts tamoxifen resistance, we performed MTT assays over a period of 7 days with V1 and O2 cell lines (Figure 6A). O2 cells were more resistant to tamoxifen than V1 cells on days 1–7 (*p* < 0.0001). Pairwise comparative adjusted means and 95% confidence intervals are shown. The O2 means are consistently higher than the V1 means, with the only overlap in confidence intervals occurring on Day 7. Factorial analysis of covariance showed that the Condition x Day interaction was not statistically significant (*p* = 0.33). The lack of interaction between Condition and Day are apparent in Figure 6A, where the time trends are very similar for the O2 and V1 conditions.

Real-time PCR was used to determine the levels of *BCAR1* mRNA, a marker of tamoxifen resistance [19], comparing V1 and O2 cells. As can be seen from Figure 6B, *BCAR1* expression was almost two fold higher in O2 than in V1 cells (*p* < 0.01).

## DISCUSSION

By a number of different measures, MCF-7 cells overexpressing *BP1* were more aggressive: they grew in the absence of serum, formed larger colonies in soft agar, were relatively more infiltrative in an invasion assay and could form tumors in mice without external estrogen supplementation. This result was consistent with our earlier findings that *BP1* positive breast tumors have a higher proliferation rate than *BP1* negative tumors [9]. We also demonstrated in our earlier study that overexpression of *BP1* significantly enhanced cell proliferation and metastatic potential in ER-negative Hs578T cells [15].

**Figure 7:**
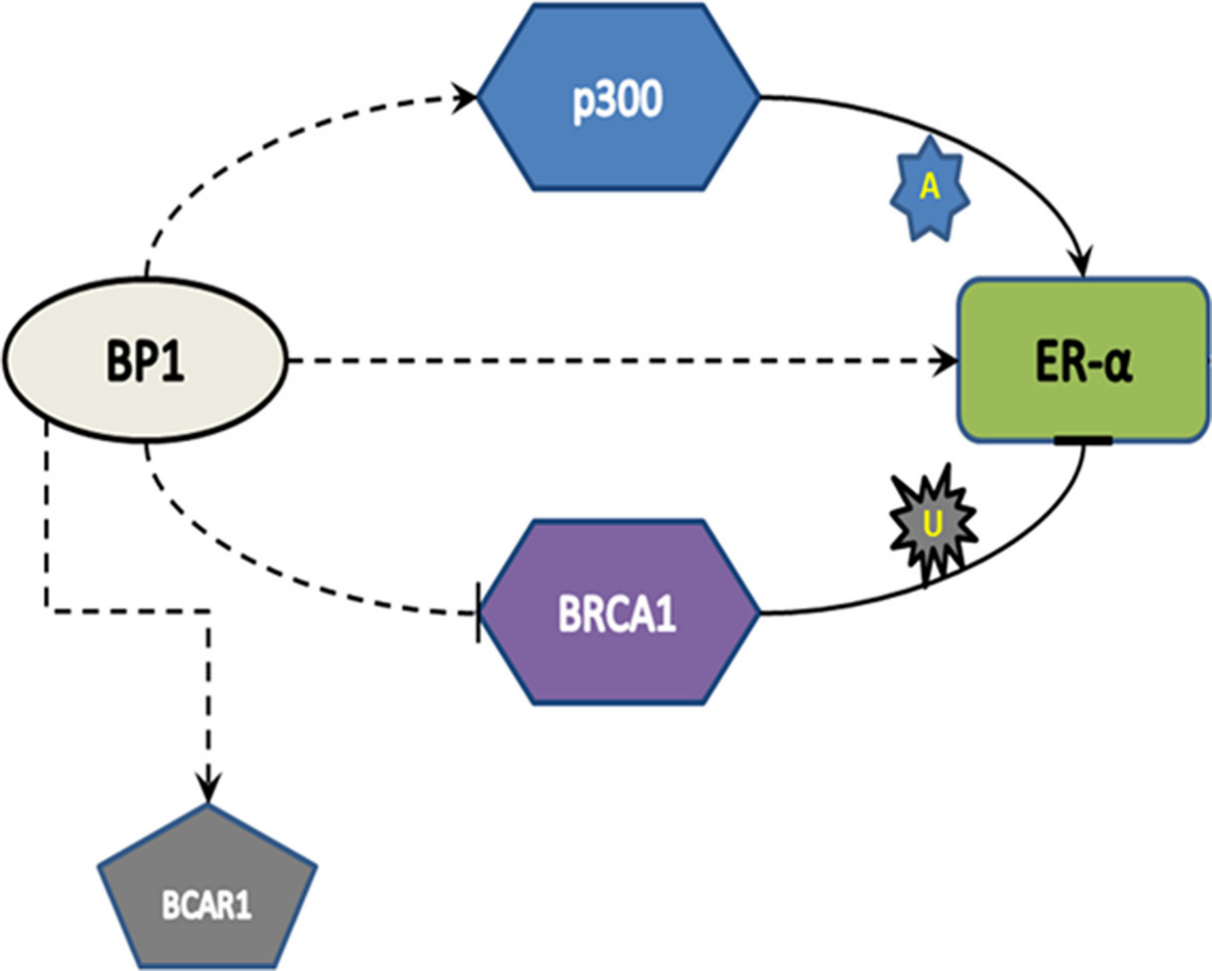
Model of *BP1* regulation of *ER* Dotted lines indicate a transcriptional mechanism, while solid lines indicate translational control (A: acetylation, U: Ubiquitination). *BP1* binds to and activates *EP300*; *p300* protein, which acetylates and “rescues” *ER* from *BRCA1*-mediated ubiquitination, is then up-regulated. Simultaneously, *BP1* binds and transcriptionally activates *ER*. The increased *ER* protein expression and stability result in increased tamoxifen resistance.

The observation that tumors developed in mice without estrogen supplementation was intriguing. Mechanisms of estrogen independence/tamoxifen resistance are not yet fully understood. Evidence in the literature points out that tumors acquire tamoxifen resistance in variety of ways, including but not limited to constitutive activation of *ER* by phosphorylation via increased growth factor signaling (non-genomic signaling pathway), leading to ligand independent activation of *ER* and thus insensitivity to estrogen, increased expression of co-activators, conversion of *ER* positive cells to *ER* negativity, lack of the 46 kDa *ER* protein, presence of the 36 kDa *ER* protein [20–24] or inappropriate increases in *ER* protein levels via increased stability [25–28]. Tamoxifen resistance/estrogen independence in *BP1* overexpressing cells is due in part to increased stability. Also, *ER*, *p300* and *BRCA1* share a dynamic relationship, with *BRCA1* inhibiting *ER* via ubiquitination, and *p300* competitively stabilizing *ER* via acetylation [17, 29, 30]. Here we found that *BP1* activates *EP300*; it is known that *BP1* represses *BRCA1* [6]. Thus, *BP1* increases *ER* protein levels by both (i) a direct mechanism: *BP1* transcriptionally upregulates *ER* and (ii) an indirect mechanism: *BP1* transcriptionally upregulates *p300*, thus aiding increased stability of *ER* (Figure 7). We also demonstrate that cells overexpressing *BP1* are more resistant to tamoxifen and express the tamoxifen resistant marker *BCAR1*; we have found a consensus *BP1* binding site in *BCAR1*, suggesting that *BP1* may directly regulate *BCAR1*.

Other HB genes have been implicated in an altered response to tamoxifen. Breast cancer cells overexpressing *HOXB7* or HOXB13 show repression of the estrogen receptor leading to estrogen independence and resistance to tamoxifen [31, 32]. In contrast, here we show that *BP1*, also a HB gene, has the ability to confer tamoxifen insensitivity by both transcriptionally upregulating and stabilizing *ER* protein.

Our previous data demonstrated that the frequency of *BP1* positivity, and the distribution and intensity of *BP1* expression, increased with the progression of tumor development (normal^→^ hyperplasia^→^ *in situ*^→^ invasive), from a few randomly distributed *BP1* positive cell clusters in normal controls to the vast majority of cells in the invasive tumors showing distinct *BP1* immunoreactivity [9]. Other indicators of relative aggressive behavior are associated with *BP1* expression in breast tumors. *BP1* positivity is associated with (i) high tumor grade [33], a classical indicator of tumor aggressiveness [34]; (ii) tumors of African American women [8], known to show relative aggressive clinical behavior and associated with increased mortality [35, 36]; (iii) inflammatory breast cancer (IBC), a form of breast cancer with poor survival [37, 38], where we found that 100% of IBC tumors were *BP1* positive, as well as nine lymph nodes from *BP1* positive, metastatic IBC tumors [39]; (iv) increased expression of the proliferation marker *Ki67* in *BP1* positive breast and prostate tumors [9, 40]; (v) decreased apoptosis due to increased expression of *BCL-2* [12]; (vi) increased expression of *VEGF* in *BP1* positive ovarian tumors (*BP1* is called *DLX4* in Hara et al. [41]). ChIP-on-chip (Chromatin Immunoprecipitation on microarray promoter chip) combined with expression microarray studies have demonstrated that *BP1* may regulate VEGF expression in Hs578T breast cancer cells as well [42]; (vii) epithelial to mesenchymal transition [43]. *BP1* is expressed in a high percentage of *ER* negative tumors as well [8]. Here we show that *BP1* overexpression correlates with increased metastatic potential, larger tumor size, less dependence on growth factors and resistance to tamoxifen treatment. A picture of the possible clinical importance of *BP1* is now emerging.

Based on gene expression profiles, attempts have been made to classify breast tumors, with luminal A being most common subtype of breast cancer representing almost 50–60% of all diagnosed cases [44]. Luminal A tumors are generally characterized by low expression of the proliferation marker Ki67, sustained expression of *ER, PR* and *BCL-2* and generally low *HER2* expression [44, 45]. These types of tumors tend to have a more favorable tumor biology, good prognosis, low histological grade, respond well to tamoxifen/endocrine therapy and form smaller tumors [45]. Luminal B tumors constitute between 10–20% of all breast cancer cases. There are a number of characteristics in common between Luminal B tumors and *BP1* positive tumors, as illustrated in this paper: (a) increased expression of proliferation genes, such as *Ki67* [9, 44, 45]; (b) *ER*+, *HER2* +/– and *PR*+/– in Luminal B tumors and frequent (73%) *BP1* positivity in *ER*+ tumors [8, 44, 45]; (c) poor prognosis and high grade [33, 44, 45]; (d) reduced sensitivity to tamoxifen ([44, 45] and shown here associated with *BP1* levels) and (e) larger tumors ([44, 45] and shown here for *BP1*). Therefore, we speculate that *BP1* overexpression in Luminal A subtype cells can “propel” those cells to display more Luminal B-like characteristics. Further study with clinical patient samples is necessary to determine whether *BP1* is a biomarker for Luminal B type tumors.

Our current data, along with previously published data, suggest that not only does *BP1* expression increase with breast tumor progression but it is also involved in regulating gene expression patterns in tumors, driving them towards a more aggressive subtype. In particular, increased *BP1* expression is associated with larger tumors, increased invasiveness of MCF-7 cells, and possibly with increased resistance to tamoxifen. Future clinical studies will provide important insights on *BP1* as a biomarker and potential therapeutic target in *ER* positive breast cancer.

## MATERIALS AND METHODS

### Cell lines and viability assays

MCF-7 cells and derivatives that overexpress *BP1* were described [12]. MCF-7 cells, as well as the empty vector and *BP1* overexpressing MCF-7 cell lines, were maintained in RPMI 1640 containing 10% FBS and 1% penicillin/streptomycin (P/S), supplemented with 500μg/ml of G418. For viability assays, 2000 cells/well were seeded in triplicate in a 96-well plate and allowed to attach overnight. Media was replaced with serum-free RPMI 1640 the next day and was changed on day 3. For tamoxifen assays, 2000 cells/wells were seeded in a 96-well plate and allowed to attach overnight. The media was replaced with phenol red free RPMI 1640 containing 5% charcoal stripped serum (CSS) and 1% P/S. The media was changed after 24 hours and 3μM tamoxifen was added (Sigma, St. Louis, MO, USA). Tamoxifen containing media was replaced every 48 hours and readings were taken from day 1 to day 7. Growth was measured by the 3-(4,5-dimethylthiazol-2-yl)-(2,3-diphenyltetrazolium) bromide dye conversion assay (Sigma) at 570 nm. T47D cell lines were grown and maintained in RPMI 1640 containing 10% FBS and 1% penicillin/streptomycin.

### Cell growth in soft agar

12,500 cells/ml were suspended in 0.3% agar supplemented with DMEM and 10% FBS and layered over 1ml of a 0.8% agar/medium base [46]. Cells were allowed to grow over a 14-day period; colonies were stained with nitroblue tetrazolium and counted using an Artek 880 colony counter.

### Invasion assays

The cell invasive capacity was estimated using BioCoat Matrigel Invasion Chambers (8 mm pores, 24 wells) (BD Bioscience, Bedford, MA, USA) as described previously [15]. In brief, cells stably transfected with an empty vector or with *BP1* cDNA plasmids were resuspended in serum-free DMEM medium (2.5 × 10^5^ cells/ml) and seeded in the top chamber of pre-wet inserts. After 48 h incubation, cells that migrated to the bottom surface of the insert were stained with Diff-Quick staining solution and quantified. The number of migrating cells was determined by counting five non-overlapping random fields on each chamber; four to five chambers were counted for each experimental point.

### Tumor formation in mice

The use of mice in this study was approved by the NIH Institutional Animal Care and Use Committee (IACUC). All procedures were conducted in accordance with the NIH Guide for the Care and Use of Laboratory Animals. Athymic nude mice were maintained on a 12 hr light/12 hr dark schedule with free access to laboratory chow and water. 2 × 10^6^cells were injected into the cleared mammary fat pads of 4–6 week old female athymic nude mice [47]. At the same time, some of the mice were also implanted subcutaneously with a 10 mg cholesterol-based pellet containing 0.72 mg of 17-β-estradiol (Innovative Research of America, Sarasota, FL, USA). Tumors were measured at the indicated times in two dimensions using calipers. At sacrifice, tumors were either frozen or fixed in 10% normal buffered formalin.

### Clinical samples

Human breast tumor samples were obtained from The George Washington University Department of Pathology with IRB approval. Breast tissue samples 0.5– 1.0 cm in diameter were obtained from frozen surgical resection specimens and characterized pathologically.

### Electrophoretic mobility shift assays (EMSA) assays

EMSA was performed as described earlier [6, 7]. Complementary sequences in the first intervening sequence (IVS1) of the *ER* were annealed and 5′-end-labeled with ^32^P-ATP using T4 kinase (Invitrogen, Grand Island, NY, USA). The Wheat Germ Coupled Transcription /Translation kit (Promega, Madison, WI, USA) was used to generate *BP1* protein from the plasmid pGEM7 containing the *BP1* open reading frame. Unlabeled competitor oligonucleotides were added at 500X or 1000X molar excess to the binding reactions. The following DNA sequences were used as probes: *ER*:

5′-GGCAAAATGCAGCTCTTCCTATATGTATAC CCTGAATCTC-3′; negative control (NC):

5′-TCTTAGAGGGAGGGCTGAGGGTTTGAAG TCCAACTCCTAAGCC-3′.

### Chromatin-immunoprecipitation (ChIP) assays

The *BP1* consensus binding site (5′-WTCWATATG-3′) on *EP300* [7] was predicted using the CISTER program [48]. Primers, flanking the putative *BP1* binding site, were designed using primer3plus [49] tool and verified by primer-BLAST [50]. The primers used for ChIP assay are as follows: 5′-GGAGCATCCTCAGATTTTGG-3′ (*EP300*-Forward) and 5′-TGCCTTAACTATCTGCTGATTTTC-3′ (*EP300*-Reverse). The ChIP protocol was performed using the Millipore ChIP kit (Millipore, Billerica, MA, USA) as described previously [6, 42]. Briefly, *BP1*-overexpressing MCF-7 or T47D cells were crosslinked at 80% confluence in 10 mL RPMI 1640 media with 1% formaldehyde for ten minutes at 37ºC. Cells were washed twice with cold PBS containing 1x complete mini protease inhibitor (Roche Applied Science, Indianapolis, IN, USA), lysed in SDS lysis buffer and incubated for fifteen minutes on ice. Chromatin was sheared by sonication for 10 pulses, twice. Equal amounts of DNA were used as test and negative controls, and 1 μg was used as the input control. 10 μg of *BP1* antibody (Bethyl Labs, Montgomery, TX, USA) or equal amounts of normal rabbit IgG (Cell signaling, Danvers, MA, USA ) were added along with salmon sperm DNA/Protein G PLUS/Protein A agarose beads, and incubated overnight at 4C. Further isolation and purification of the precipitated DNA was done according to Millipore's protocol. Isolated DNA was resuspended in Tris-EDTA buffer for use in PCR. Platinum superscript master mix was used with a 10 μM final concentration of each primer and 1 μg of DNA as input or 5 μl of isolated/precipitated DNA in a final reaction volume of 45 μl. PCR was run for 40 cycles. The PCR product was analyzed on a 2% agarose gel and visualized.

### Real-time PCR assays

Total RNA was prepared using the RNeasy Mini Kit (Qiagen Inc, Valencia, CA. USA). RNA samples underwent DNase I treatment (Promega, Madison, WI, USA) prior to first-strand cDNA synthesis with random hexamer primers using the Superscript II First-Strand cDNA Synthesis System (Invitrogen, Grand Island, NY, USA). Real-time PCR was carried out using the ABI 7300 model sequence detection system (Applied Biosystem, Foster City, CA, USA) with SYBR Green I PCR Mastermix (BioRad, Hercules, CA, USA). Primers for ER and *EP300* were published [51–53]. Primers for *BP1* were designed using Primer-BLAST and are as follows: 5′-CCTCCCCCAATTTGTCCTACTC-3′ (forward) and 5′-GGTTGCTGGCAGGACAGGTA-3′ (reverse). The amplification program included an initial denaturation at 95°C for 10 min., followed by 40 cycles of a two-stage PCR consisting of 95°C for 15s and 60°C for 1 min. Specificity for PCR amplifications was verified by observing a single peak dissociation curve for each gene. One microliter of the reverse transcribed cDNA was used for each real-time PCR reaction and all reactions were performed in triplicate. The expression values of genes from different samples were calculated by normalizing with 18S RNA and relative quantitation values were plotted.

### Immunoblotting assays

Cells were grown to 70%–80% confluency and proteins extracted as previously described [12]. 30–65 μg of proteins were loaded on 4–20% SDS-PAGE gels (Bio-Rad, Hercules, CA, USA) and transferred to nitrocellulose membranes. Blots were probed overnight with rabbit anti-*BP1* (Bethyl Labs, Montgomery, TX, USA) at a 1:5000 dilution, with rabbit antiER-α (Bethyl labs, Montgomery, TX, USA) at a 1:1000 dilution, mouse anti-*p300* (Millipore, Billerica, MA, USA) at a 1:500 dilution, or mouse anti-beta-actin (Sigma, St. Louis, MO, USA) at a 1:5000 dilution. After washing, blots were incubated with either horseradish peroxidase-linked goat anti-mouse (1:2500 dilution) or goat anti-rabbit secondary antibodies (1:15,000 dilution). Signals were detected using SuperSignal West Dura Extended Duration Substrate (Pierce, Rockford, IL, USA).

### Statistical analyses

For analysis of mouse data, due to the smaller sample sizes, data were first pooled for the mice receiving the two *BP1*- overexpressing cell lines when tested against the control mice. For the test of colony size differences, results are based on the pooled data from six independent replications (two experimental replications of three wells each). For tamoxifen experiments, in order to adjust for any ethanol (EtOH) effects on optical density, scores were calculated by subtraction (subtracting mean optical density under the EtOH condition for each day for O2 and V1 from the tamoxifen condition scores) and by ratio (dividing the tamoxifen condition scores by the mean optical density under the EtOH condition for each day for O2 and V1). The ratio adjustment method was used for all subsequent analyses because of greater homoscedasticity and reasonable evidence of normality. The statistical test of mean differences on study condition (O2 vs V1), follow-up day, and Condition x Day interaction was by factorial analysis of covariance. The qPCR data for *ER* and *p300* were analyzed by one-sample *t*-test.
